# Therapy with atorvastatin versus rosuvastatin reduces urinary podocytes, podocyte-associated molecules, and proximal tubule dysfunction biomarkers in patients with type 2 diabetes mellitus: a pilot study

**DOI:** 10.1080/0886022X.2016.1254657

**Published:** 2016-11-13

**Authors:** Adrian Vlad, Mihaela Vlad, Ligia Petrica, Sorin Ursoniu, Florica Gadalean, Roxana Popescu, Daliborca Vlad, Victor Dumitrascu, Gheorghe Gluhovschi, Cristina Gluhovschi, Silvia Velciov, Flaviu Bob, Petru Matusz, Alina Secara, Anca Simulescu, Dragos Catalin Jianu

**Affiliations:** a Department of Diabetes and Metabolic Diseases, “Victor Babes” University of Medicine and Pharmacy, Timisoara, Romania;; b Department of Endocrinology, “Victor Babes” University of Medicine and Pharmacy, Timisoara, Romania;; c Department of Nephrology, “Victor Babes” University of Medicine and Pharmacy, Timisoara, Romania;; d Department of Public Health Medicine, “Victor Babes” University of Medicine and Pharmacy, Timisoara, Romania;; e Department of Cellular and Molecular Biology, “Victor Babes” University of Medicine and Pharmacy, Timisoara, Romania;; f Department of Pharmacology, “Victor Babes” University of Medicine and Pharmacy, Timisoara, Romania;; g Department of Anatomy, "Victor Babes" University of Medicine and Pharmacy, Timisoara, Romania;; h Department of Nephrology, “Pius Brinzeu”, County Emergency Hospital, Timisoara, Romania;; i Department of Neurology, “Victor Babes” University of Medicine and Pharmacy, Timisoara, Romania

**Keywords:** Atorvastatin, diabetic nephropathy, urinary podocytes, proximal tubule biomarkers, advanced glycation end-products

## Abstract

**Background:** Diabetic nephropathy is a severe complication of Type 2 diabetes. Tubular lesions may play an important role in its early stages. The aim of our study was to determine if atorvastatin protects the podocytes and the proximal tubule in patients with Type 2 diabetes.

**Methods:** A total of 63 patients with Type 2 diabetes completed this 6-months prospective pilot study. They were randomized to continue rosuvastatin therapy (control group) or to be administered an equipotent dose of atorvastatin (intervention group), and were assessed regarding urinary podocytes, podocyte-associated molecules, and biomarkers of proximal tubule dysfunction.

**Results:** The patients from the intervention group presented a significant reduction in podocyturia (from 7.0 to 4.0 cells/ml, *p* < .05), urinary nephrin (from 1.7 to 1.3 mg/g, *p* < .001), urinary vascular endothelial growth factor (from 262.8 to 256.9, *p* < .01), urinary alpha_1_-microglobulin (from 10.0 to 8.3 mg/g, *p* < .01), urinary kidney injury molecule-1 (from 139.5 to 136.3 ng/g, *p* < .001), and urinary advanced glycation end-products (from 112.6 to 101.3 pg/ml, *p* < .001). Podocyturia correlated directly with the podocyte damage biomarkers, proximal tubule dysfunction biomarkers, albumin to creatinine ratio, and advanced glycation end-products, and inversely with the glomerular filtration rate.

**Conclusions:** In patients with Type 2 diabetes, atorvastatin exerts favorable effects on the kidney. There is a correlation between the evolution of the podocytes and of the proximal tubule biomarkers, supporting the hypothesis that the glomerular changes parallel proximal tubule dysfunction in the early stages of diabetic nephropathy.

## Introduction

The number of cases of diabetes mellitus is steadily increasing worldwide. Patients may develop in time chronic complications that represent an important part of the “diabetes burden”. Among these, diabetic nephropathy is of utmost importance. In developed countries, diabetes mellitus is the leading cause of end-stage renal disease,^1^ and the risk of death due to renal disease is estimated to be 3 times higher in the population with diabetes.^2^

The mechanisms that lead to the development of diabetic nephropathy are not completely understood. The classical concept considers that the severity of the defects in the glomerular filtration barrier determine the magnitude of urinary albumin loss.^3^ To date, there is a paradigm shifting regarding the mechanisms of albuminuria in the early stages of diabetic nephropathy, from the aforementioned glomerular theory to a tubular theory, which states that the occurrence of albuminuria is mainly related to the proximal tubule dysfunction rather than to an increased leakiness of the glomerular filter.^4–6^

The podocytes are located on the outer aspect of the glomerular basement membrane. Their detachment and loss by urinary excretion, together with derangements in the architecture of the glomerular endothelial cells, may contribute to both the initiation and the progression of diabetic nephropathy.^7^^,^^8^

Lipids are important components of the podocytes and lipid accumulation was described in several glomerular diseases. A better understanding of their biology in podocytes might lead to the development of new therapeutic approaches to primary and secondary glomerulopathies, including diabetic nephropathy.^9–11^

The hydroxymethylglutaryl coenzyme A (HMG-CoA) inhibitors or statins are drugs that inhibit endogenous synthesis of cholesterol, by blocking the rate limiting step in its synthesis. Consequently, the intracellular concentration of cholesterol decreases, and the expression of LDL-receptors on cell surface increases, which results in an enhanced extraction of LDL cholesterol from the blood. In addition, statins are credited with pleiotropic effects.^12^^,^^13^ Statins are different regarding their chemical structure, binding site, affinity for HMG-CoA reductase, and lipophilicity. According to the last characteristic, they may be considered hydrophilic (rosuvastatin, pravastatin, pitavastatin) or lipophilic (atorvastatin, lovastatin, simvastatin, fluvastatin). The more lipophilic statins tend to achieve higher levels of exposure in non-hepatic tissues, while the hydrophilic ones tend to be more hepatoselective. The diffusion of statins into the extrahepatic tissues increases the probability of intolerance to these drugs, but, in addition, it may exert protective effects in the organs affected by diabetes, such as the kidney.^13–17^ The effects of different statins on the podocytes have been studied in various types of glomerulopathies, including diabetic nephropathy, in experimental and clinical setting. Their influence on these cells is probably different, depending on the solubility of the drug, but the information is scarce concerning this issue.^18–21^

The aim of our study, conducted on patients with Type 2 diabetes mellitus, was to determine the influence of a lipophilic statin (i.e., atorvastatin) on the podocytes and proximal tubule dysfunction, after switching from a hydrophilic one (i.e., rosuvastatin). The effects of the statins were assessed by the level of podocyte excretion in the urine, as well as by the biomarkers of podocyte damage and of proximal tubule dysfunction. The relationship of these changes with urinary advanced glycation end-products (AGE) was also studied.

## Materials and methods

### Study groups

This was a prospective randomized pilot study, conducted in patients with Type 2 diabetes mellitus within a period of 6 months. The inclusion criteria were represented by duration of diabetes higher than 5 years, stable therapy with antidiabetic drugs, and good blood pressure control with a regimen including an angiotensin-converting enzyme inhibitor or an angiotensin receptor blocker, and stable therapy with rosuvastatin. The exclusion criterion was represented by a diagnosis of non-diabetic chronic kidney disease. In order to accurately diagnose a specific podocitopathy, renal biopsy is required. The patients included in this study had a long duration of diabetes mellitus (>5 years), the urinary sediment was without red blood cells, their renal function was stable within the previous years and during the follow-up period, and many presented other diabetic complications, such as retinopathy and peripheral neuropathy. They did not have indication for renal biopsy, thus this investigation was not performed.

**Figure 1.**
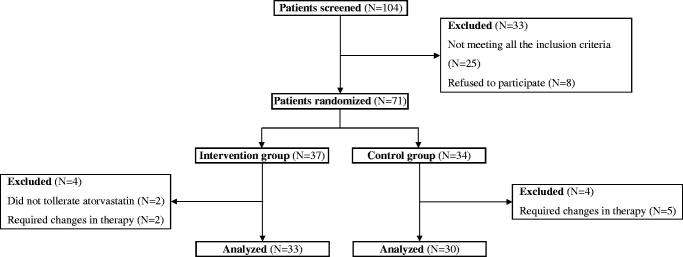
Flow-chart regarding the enrollment of the study participants.

In total, 71 out of 104 patients who attended the Diabetes and Metabolic Diseases or the Nephrology Outpatient Departments from January 2014 through June 2014 and who fulfilled all the criteria for the study were randomly assigned (Figure 1) to be administered an equipotent dose of atorvastatin (intervention group) or to continue therapy with rosuvastatin (control group). No other therapeutic intervention was recommended. Out of these, 33 patients from the intervention group (7 normoalbumunuric, 11 microalbuminuric, and 15 macroalbuminuric), 10 men (30.3%) and 23 women (69.7%), with a mean age of 58.4 ± 6.5 years (range 47–75 years) and a mean disease duration of 10.5 ± 3.6 years (range 5–21 years), and 30 patients from the control group (7 normoalbumunuric, 10 microalbuminuric, and 13 macroalbuminuric), 11 men (36.7%) and 19 women (63.3%), with a mean age of 60.0 ± 7.6 years (range 46–72 years) and a mean disease duration of 10.0 ± 3.1 years (range 5–18 years), completed the study and were analyzed.

The subjects were assessed at baseline and after 6 months. The biologic work-up included routine biochemical parameters; urinary albumin to creatinine ratio (UACR); urinary nephrin, urinary vascular endothelial growth factor (VEGF), and urinary podocyte count, as markers of podocyte damage; urinary alpha_1_-microglobulin and urinary kidney injury molecule-1 (KIM-1), as biomarkers of proximal tubule dysfunction; and urinary AGE. The biomarkers were determined from specimens frozen at −80 °C and thawed before the assay. The screening for other diabetes-related complications (retinopathy, peripheral neuropathy, and cardiovascular diseases) was performed at baseline, as part of the routine checkup. It was not repeated at the end of the study, as the follow-up period was too short to induce significant changes in these parameters.

Chronic kidney disease was defined according to the KDIGO Guideline for the Evaluation and Management of Chronic Kidney Disease.^22^

### Laboratory tests

*Albuminuria* was determined by immunonephelometry, with N Antiserum to Human Albumin (Siemens Healthcare Diagnostics, Marburg, Germany), in the second urine specimen from the morning. The antiserum yielded a within-run coefficient of variation (CV) of 2.2% and a total CV of 2.6%, with a mean of 79 mg/l. Urine cultures were negative for bacteriuria in all patients. UACR was then calculated, and the cut off limits for microalbuminuria and macroalbuminuria were considered as 30 mg/g, and 300 mg/g, respectively.

*Cultures of urinary podocytes* were performed as described elsewhere.^23^^,^^24^ Midstream urine samples of 30 ml were collected in sterile tubes and centrifuged (5 min, 700 *g*). The pelleted cellular material was washed twice with PBS, suspended in RPMI medium supplemented with 10% fetal bovine serum, insulin-transferrin-selenium G (Gibco, New York, NY, cat. number 41400045) and 1% penicillin/streptomycin (Life Technologies, Carlsbad, CA, cat. number 15140–122), and cultured on flasks coated with type I collagen (Gibco, New York, NY, cat. number A10483–01). Samples were incubated overnight at 37 °C with 5% CO_2_. After 12 h, the cells were detached with trypsin, suspended in PBS, and centrifuged (5 min, 700 *g*). They were fixed thereafter with paraformaldehyde in PBS (30 min at room temperature, and 10 min in methanol at −20 °C), followed by permeabilization with 0.2% Triton X-100 in PBS (10 min). Nonspecific binding sites were blocked with 2% FBS and 2% BSA in PBS overnight. The slides were incubated with the primary antibody PCX (Podocalyxin Mouse Monoclonal Antibody—clone 3D3—cat. number 39–3800, Invitrogen, Carlsbad, CA) for 2 h at room temperature. After washing with PBS, the slides were incubated with the secondary antibody (Life Technologies, Carlsbad, CA, fluorescein goat anti-mouse IgG-(H + L), F2761) for 1 h, examined by immunofluorescence microscopy and counted. Urinary podocytes were expressed as cells/ml.

*Nephrin* was measured in the second morning urine specimen with the aid of a human NPHN antibody (ELISA kit, cat. number E-EL-H1901, Elabscience Biotech Co. Ltd, Wuhan, Hubei Province, China). The minimum detectable dose of Human NPHN was 0.1 ng/ml. The detection range was 0.16–10 ng/ml. The CV at repeated tests was <10%.

*VEGF* was determined in the second morning urine specimen, using a human VEGF antibody (ELISA kit, cat. number ab100663, Abcam, Cambridge, MA). The minimum detectable dose of VEGF was <10 pg/ml. The intra- and inter-assay reproducibility was <10% CV, and <12% CV, respectively.

*Alpha_1_-microglobulin* was evaluated in the second morning urine specimen, with the aid of the N α1-microglobulin kit (Siemens Healthcare Diagnostics, Marburg, Germany) through particle-enhanced immunonephelometry. The reference interval was 12 mg/l or 0.07–5 mg/g creatinine. The intra-assay precision was 2.9–5.2% CV, while the inter-assay precision was 7.4–13.2% CV.

*KIM-1* was assessed in the second morning urine specimen by KIM-1 ELISA test kit for the detection of KIM-1 in human urine (cat. number H-RENA-E-001, Bio Assay Works, Ijamsville, MD). A human KIM-1 antibody was utilized and the detection level was set at urinary KIM-1 < 0.150 ng/ml.

*Urinary AGE peptides* were assessed in two 24 h urine samples by the ELISA method with human AGE ELISA kit (E01A0002, Shanghai Blue Gene Biotech Co., Shanghai, China). This assay contains polyclonal antibodies which assess both high and low molecular protein-bound AGE species. No significant interference between AGE and analogs was observed. The sensitivity of this assay was 1.0 pg/ml.

### Statistical analysis

Clinical and biological data are presented as medians and interquartile ranges (for variables with skewed distribution), as mean values ± SD (for variables with symmetric distribution), or as numbers and percentages. The differences between subgroups were analyzed with the Wilcoxon matched paired test, the Kruskal–Wallis test, the paired or unpaired Student’s *t*-test, or the Fisher’s exact test. The biological data with skewed distribution were logarithmically transformed and a correlation matrix was performed. The relationship between variables was assessed using *R* values.

The *p* Values for all hypothesis tests were two-sided, and statistical significance was set at *p* < .05. All analyses were conducted with Stata 9.2 (Statacorp, College Station, TX).

### Ethical issues

The Ethical Committee of the County Emergency Hospital Timisoara approved the protocol (approval number 3/5 January 2014), and every patient provided written informed consent before enrollment.

## Results

The clinical and biological characteristics of the patients who completed the study, at baseline and at the end of the follow-up interval, are shown in Table 1.

**Table 1.** Clinical and biological data of the patients from the two study groups.

	Intervention group (*N* = 33)		Control group (*N* = 30)				
Parameter	Baseline	End of follow-up	Baseline	End of follow-up	*p*^∫^	*p*^∫∫^	*p*^∫∫∫^
BMI (kg/m^2^)	34.6 ± 5.3	34.6 ± 4.7	35.3 ± 5.0	35.3 ± 5.1	NS^b^	NS^a^	NS^a^
SBP (mmHg)	142.4 ± 16.3	142.4 ± 15.7	144.8 ± 16.7	144.4 ± 16.8	NS^b^	NS^a^	NS^a^
DBP (mmHg)	81.4 ± 10.8	79.4 ± 11.8	81.0 ± 9.6	81.7 ± 9.8	NS^b^	NS^a^	NS^a^
Diabetic retinopathy (%)	54.5	–	56.7	–	NS^c^	–	–
Peripheral neuropathy (%)	51.5	–	46.7	–	NS^c^	–	–
Cardiovascular diseases (%)	39.4	–	40	–	NS^c^	–	–
Hb (g/dl)	12.1 ± 1.5	11.9 ± 1.3	12.1 ± 1.8	12.1 ± 1.8	NS^b^	NS^a^	NS^a^
Fasting glycemia (mg/dl)	138.0 (118.0–166.0)	141.0 (121.0–170.0)	146.0 (130.0–178.0)	144.5 (120.0–170.0)	NS^d^	NS^d^	NS^d^
HbA_1c_ (%)	7.7 ± 1.1	7.7 ± 1.1	8.1 ± 1.6	8.0 ± 1.7	NS^b^	NS^a^	NS^a^
HbA_1c_ (mmol/mol)	60.2 ± 11.7	60.9 ± 12.1	65.0 ± 17.6	64.4 ± 18.5	NS^b^	NS^a^	NS^a^
Serum creatinine (mg/dl)	1.3 ± 0.4	1.3 ± 0.5	1.3 ± 0.4	1.3 ± 0.5	NS^b^	NS^a^	NS^a^
eGFR (ml/min/1.73m^2^)	55.2 ± 20.0	55.3 ± 21.5	58.5 ± 22.9	59.2 ± 24.4	NS^b^	NS^a^	NS^a^
Serum cholesterol (mg/dl)	208.0 ± 35.7	205.7 ± 34.2	211.1 ± 32.8	209.0 ± 33.8	NS^b^	NS^a^	NS^a^
Triglycerides (mg/dl)	150.0 (125.0–188.0)	161.0 (132.0–178.0)	154.5 (121.0–202.0)	159.0 (125.0–196.0)	NS^e^	NS^d^	NS^d^
hsCRP (mg/dl)	12.8 (10.0–24.2)	13.9 (9.3–25.0)	14.2 (8.8–29.3)	14.5 (8.5–28.7)	NS^e^	NS^d^	NS^d^
UACR (mg/g)	117.0 (46.3–853.9)	110.7 (40.1–807.5)	124.0 (43.0–839.4)	112.1 (36.7–823.7)	NS^e^	NS^d^	NS^d^
Urinary podocytes (cells/ml)	7.0 (5.0–10.0)	4.0 (1.0–9.0)	6.0 (4.0–9.0)	6.5 (4.0–8.0)	NS^e^	<.05^d^	NS^d^
– Normoalbuminuria	4.5 (3.5–5)	5.0 (1.0–4.5)	4.0 (2.5–4.5)	4.0 (3.0–4.5)			
– Microalbuminuria	7.0 (6.0–8.5)	2.0 (0.5–3.5)	6.5 (5.3–8.8)	7.0 (6.0–8.0)			
– Macroalbuminuria	10.0 (5.5–11.0)	8.0 (5.5–9.0)	8.0 (6.0–10.0)	7.0 (5.0–9.0)			
Urinary nephrin/creat (mg/g)	1.7 (1.0–6.9)	1.3 (0.5–4.9)	1.7 (0.9–7.1)	1.8 (1.0–7.0)	NS^e^	<.001^d^	NS^d^
– Normoalbuminuria	0.2 (0.2–0.2)	0.2 (0.1–0.2)	0.2 (0.2–0.2)	0.2 (0.2–0.2)			
– Microalbuminuria	1.5 (1.1–1.7)	1.0 (0.7–1.1)	1.5 (1.1–1.6)	1.6 (1.2–1.7)			
– Macroalbuminuria	7.4 (6.1–10.4)	5.2 (4.3–8.9)	7.2 (6.3–10.5)	7.7 (6.1–11.1)			
Urinary VEGF/creat (ng/g)	262.8 (180.0–905.3)	256.9 (127.4–723.5)	265.3 (184.8–903.7)	267.1 (189.5–927.1)	NS^e^	<.01^d^	NS^d^
– Normoalbuminuria	171.7 (141.4–191.4)	155.1 (68.2–178.5)	171.7 (131.5–187.2)	161.4 (133.4–194.5)			
– Microalbuminuria	210.0 (158.1–253.5)	163.5 (102.9–184.0)	230.9 (174.3–249.4)	238.8 (170.0–259.1)			
– Macroalbuminuria	953.8 (716.0–1449.8)	976.5 (532.5–1321.2)	1013.9 (818.5–1369.7)	953.1 (884.0–1339.0)			
Urinary alpha_1_/creat (mg/g)	10.0 (7.0–56.7)	8.3 (5.7–42.0)	9.9 (7.1–55.8)	9.0 (6.5–52.9)	NS^e^	<.01^d^	NS^d^
– Normoalbuminuria	6.5 (6.2–6.7)	6.5 (4.7–6.7)	6.8 (6.3–6.9)	6.5 (6.2–6.8)			
– Microalbuminuria	9.1 (7.8–9.5)	6.6 (5.7–7.1)	8.9 (8.4–9.7)	9.0 (8.0–9.7)			
– Macroalbuminuria	57.1 (42.1–66.9)	45.1 (39.7–57.4)	59.3 (50.9–70.9)	53.0 (26.0–59.9)			
Urinary KIM-1/creat (ng/g)	139.5 (110.0–687.9)	136.3 (78.0–646.8)	151.1 (108.6–818.6)	151.8 (106.7–808.5)	NS^e^	<.001^d^	NS^d^
– Normoalbuminuria	100.4 (98.3–112.0)	86.3 (42.4–106.0)	107.8 (98.9–109.1)	105.3 (99.9–107.3)			
– Microalbuminuria	120.0 (84.6–134.6)	87.0 (61.4–101.7)	139.1 (101.3–148.4)	133.6 (98.1–147.3)			
– Macroalbuminuria	765.6 (518.7–858.9)	711.9 (496.4–865.1)	842.2 (750.0–940.4)	867.7 (723.2–996.8)			
Urinary AGE (pg/ml)	112.6 (70.8–479.5)	101.3 (69.2–466.1)	118.3 (68.6–483.0)	121.9 (72.1–494.8)	NS^e^	<.001^d^	NS^d^

BMI: body mass index; SBP: systolic blood pressure; DBP: diastolic blood pressure; Hb: hemoglobin; HbA_1c_: glycated hemoglobin; eGFR: estimated glomerular filtration rate; hsCRP: high sensitive C reactive protein; UACR: urinary albumin to creatinine ratio; nephrin/creat: nephrin to creatinine ratio; VEGF/creat: vascular endothelial growth factor to creatinine ratio; alpha_1_/creat: alpha_1_-microglobulin to creatinine ratio; KIM-1/creat: kidney injury molecule-1 to creatinine ratio; AGE: advanced glycation end-products.

The values are expressed as mean ± SD or median (25–75 percentiles). Comparison between groups were performed by

a paired *t* test.

b Unpaired *t* test.

c Fisher’s exact test.

d,e Wilcoxon’s matched paired test or the Kruskal–Wallis test.

The statistical significance was calculated between the baseline values of the intervention and the control group (*p*^∫^), the baseline and final values of the intervention group (*p*^∫∫^), and the baseline and final values of the control group (*p*^∫∫∫^).

In all the 63 patients that completed the study, the urine podocyte count and the levels of the biomarkers for podocyte damage and proximal tubule dysfunction were higher in patients with more severe kidney involvement.

After 6 months of atorvastatin therapy, the number of urinary podocytes decreased significantly in the intervention group, from 7.0 (5.0–10.0) cells/ml to 4.0 (1.0–9.0) cells/ml (*p* < .05), whereas it remained unchanged in the control group. The decrease in the number of the podocytes was observed in 26 patients (78.8%) allocated to atorvastatin.

In parallel with the podocyte count, the patients from the intervention group presented a decrease in the level of the podocyte damage biomarkers, nephrin and VEGF [from 1.7 (1.0–6.9) mg/g to 1.3 (0.5–4.9) mg/g, *p* < .001, and from 262.8 (180.0–905.3) ng/g to 256.9 (127.4–723.5) ng/g, *p* < .01, respectively], as well as of the proximal tubule dysfunction biomarkers, alpha_1_-microglobulin and KIM-1 [from 10.0 (7.0–56.7) mg/g to 8.3 (5.7–42.0) mg/g, *p* < .01, and from 139.5 (110.0–687.9) ng/g to 136.3 (78.0–646.8) ng/g, *p* < .001, respectively]. In addition, the level of urinary AGE decreased from 112.6 (70.8–479.5) pg/ml to 101.3 (69.2–466.1) pg/ml, *p* < .001. These biological markers did not vary significantly in the controls. Of note, the changes in the podocyte count, as well as in the podocyte and tubular damage biomarkers, seemed to be more important in the patients from the intervention group with less severe kidney damage (normo- and microalbuminuric cases), as compared to those with macroalbuminuria. However, the small size of the subgroups prevented the calculation of the statistical significance of these differences.

The rest of the studied parameters did not present significant changes within the study period in neither group. After adjustment for blood pressure and glucose control, the multivariate analysis showed a significant direct correlation between the number of urinary podocytes, and the podocyte damage biomarkers, proximal tubule dysfunction biomarkers, UACR and AGE, and a significant inverse correlation between podocyturia and eGFR (Table 2).

**Table 2.** Correlations between the biologic parameters in the intervention group.

	Podocytes	Nephrin	VEGF	Alpha_1_	KIM-1	AGE	UACR	eGFR
Podocytes	1.0000							
Nephrin	0.6252	1.0000						
VEGF	0.5673	0.8899	1.0000					
Alpha_1_	0.4467	0.8722	0.9477	1.0000				
KIM-1	0.3314	0.7826	0.8696	0.9400	1.0000			
AGE	0.3668	0.7678	0.8512	0.9195	0.9421	1.0000		
UACR	0.4605	0.9415	0.9384	0.9150	0.8604	0.8097	1.0000	
eGFR	−0.3829	–0.7831	–0.7837	–0.7453	–0.7029	–0.7635	–0.7957	1.0000

VEGF: vascular endothelial growth factor; alpha_1_: alpha_1_-microglobulin; KIM-1: kidney injury molecule-1; AGE: advanced glycation end-products; UACR: urinary albumin to creatinine ratio; eGFR: estimated glomerular filtration rate.

The values in the table represent the correlation coefficient *R*, after logarithmically transforming the biological data with skewed distribution.

## Discussion

In this work, performed in patients with Type 2 diabetes mellitus, we studied the influence on podocytes and proximal tubule dysfunction of a lipophilic statin (atorvastatin), after switching from a hydrophilic one (rosuvastatin), by analyzing the evolution of the number of podocytes in the urine, as well as of the urinary excretion of several podocyte and proximal tubule dysfunction biomarkers. We found that this therapeutic intervention diminished the urinary podocyte excretion and the level of the biomarkers studied. The beneficial effects on the UACR and the eGFR did not reach the threshold for statistical significance.

### Podocyte excretion in the urine is reduced by atorvastatin

The podocyte seems to be an important player in the development of renal involvement in patients with diabetes mellitus. Podocyte detachment and podocyte loss occur in the evolution of various glomerular diseases, including diabetic nephropathy. Urinary excretion of podocytes, as well as of podocyte biomarkers (such as VEGF or nephrin), may represent useful tools for the detection of kidney involvement and for monitoring its progression.

A number of experimental studies have described some mechanisms by which different statins act on the podocyte.^25^ The effect of the HMG-CoA reductase on the kidney has been tested in clinical setting, as well.^15^ However, little is known about the different effects of hydro- and lipophilic statins on the kidney injury in patients with Type 2 diabetes mellitus.^19^

In our study, the podocytes were counted in the urine of all subjects at baseline and by the end of the follow-up. At baseline, their number varied between subgroups and was significantly higher in the macroalbuminuric patients as compared to the normo- and microalbuminuric subjects. Our results are similar to those reported by Ye et al.,^26^ but differ from the findings of Nakamura et al.,^23^ who found no podocyte excretion in normoalbuminuric patients. The presence of the podocytes in urine even before the occurrence of microalbuminuria stresses their possible role as markers of the early stages of diabetic nephropathy.

The podocyte biomarkers analyzed in our study, nephrin and VEGF, had a similar behavior: their urinary levels paralleled the progression of the albumin excretion level, being detectable even in the normoalbuminuric subgroup. These data are in concordance with the results of other clinical and experimental studies,^27–31^ which demonstrated the utility of nephrin and VEGF as early biomarkers of diabetic nephropathy or as predictors of the development of renal insufficiency in the stage of normoalbuminuria.

Mean podocyturia decreased significantly after the therapeutic intervention, and the beneficial effects were observed in the majority of the patients (78.8%), while in the remaining subjects the number of urinary podocytes was unchanged by the end of the follow-up period. The podocyte damage biomarkers were influenced in a similar way by the switch in the statin therapy. Our results are in keeping with those reported by Takemoto et al.,^19^ who found a reduction in urinary podocyte excretion with the switch from rosuvastatin to atorvastatin. However, the number of cells/ml was higher in our study. This could be explained by the inclusion of patients with macroalbuminuria, who have significantly higher urinary podocyte excretion (they were not recruited in the aforementioned study), or by differences in the technique of measuring podocyturia.

The mechanisms by which statins could exert beneficial effects may be mediated by their influence on lipoproteins or by other effects. Lipophilic statins (such as atorvastatin) enter the membrane of various cells, including podocytes, and inhibit the excessive intracellular signaling, particularly by membrane-bound small GTPase, and, consequently, the actin cytoskeleton reorganization.^32^ Hydrophilic statins (such as rosuvastatin) do not share the same properties. In our study, the switch in statin therapy was done in equipotent doses, and the mean value of the lipid parameters did not change significantly during the follow-up period. Consequently, one can assume that the benefits of atorvastatin therapy are due to their lipophilicity, as suggested by other data from the literature.^32^

The levels of urinary AGE decreased significantly after 6 months of therapy with atorvastatin. This observation leads to the hypothesis that the detachment of podocytes and their urinary excretion, as well as the increased expression of podocyte damage biomarkers, could be AGE-mediated phenomena.

The parallel evolution of urinary podocytes, nephrin, and VEGF suggests the utility in the clinical practice of the whole panel of biomarkers, in order to accurately diagnose diabetic nephropathy in its early stages.

### Proximal tubule dysfunction biomarkers parallel podocyturia under atorvastatin therapy

Diabetic tubulopathy is an emerging entity that explains the occurrence of albuminuria in the early stages of diabetic nephropathy as a result of the impaired tubular reabsorption of albumin, rather than of its increased glomerular filtration.^6^^,^^33^

In our study, the evolution of the markers of proximal tubule dysfunction, urinary alpha_1_-microglobulin, and KIM-1, correlated significantly with podocyturia and the podocyte biomarkers. Fu et al. suggested that there is a link between the glomerular functional changes and the tubular damage: the glomerular hyperfiltration, that characterizes the early stages of diabetic nephropathy, could be a trigger for the proximal tubule dysfunction.^34^ The strong correlation found by us between the podocytes and the proximal tubule biomarkers supports this hypothesis.

The switch in the statin therapy exerted beneficial effects not only on the podocyte biomarkers, but also on the tubular ones, by decreasing urinary alpha_1_-microglobulin and urinary KIM-1. This decrease seemed to be more important in the normo- and microalbuminuric patients but, due to the small number of the subjects, statistical significance could not be calculated. The mechanisms by which statins protect the proximal tubule may be mediated by AGE,^35^ and this action may be more obvious in the early stages of diabetic nephropathy.

### Strengths and limitations of the study

Our work has several limitations. First, the sample size was small, thus the results require validation in larger cohorts. Second, the follow-up period was short, fact that could impair the statistical significance of some findings. This study was meant to be a pilot trial. Therefore, in the setting of these promising results, it should be followed by a larger study, with a longer duration.

The strength of our study is represented by the fact that it demonstrated the beneficial effects of a lipophilic HMG-CoA inhibitor, atorvastatin, on the urinary excretion of podocytes and their damage biomarkers, as well as on the excretion of proximal tubule dysfunction biomarkers. To the best of our knowledge, this is the first study to document the association between these parameters, in the setting of a switch from a hydrophilic statin to a lipophilic one.

To conclude, our study suggests the positive effects of atorvastatin on the kidney in patients with Type 2 diabetes mellitus. It reveals the correlation between the evolution of the urinary excretion of podocytes and their damage biomarkers, and of the proximal tubule biomarkers under atorvastatin therapy, supporting the hypothesis that the glomerular changes parallel proximal tubule dysfunction in the early stages of diabetic nephropathy.
